# Genome sequence and evaluation of safety and probiotic potential of *Lacticaseibacillus paracasei* LC86 and *Lacticaseibacillus casei* LC89

**DOI:** 10.3389/fmicb.2024.1501502

**Published:** 2025-01-27

**Authors:** Ting Chen, Yunjiao Zhao, Yixuan Fan, Yao Dong, Zhonghui Gai

**Affiliations:** ^1^Department of Research and Development, Wecare Probiotics Co., Ltd., Suzhou, China; ^2^College of Life Science and Technology, Huazhong Agricultural University, Wuhan, China

**Keywords:** *Lacticaseibacillus casei*, *Lacticaseibacillus paracasei*, genome sequence, probiotic potential, safety

## Abstract

**Aim:**

A comprehensive safety assessment of potential probiotic strains was essential for their application in the food industry. This article systematically evaluated the probiotic characteristics, whole-genome sequence analysis and safety of *Lacticaseibacillus paracasei* LC86 and *Lacticaseibacillus casei* LC89.

**Methods:**

Firstly, the two strains of lactic acid bacteria selected were identified. Secondly, whole-genome sequencing was performed on LC86 and LC89, and their antibiotic resistance, pathogenicity, and virulence genes were analyzed. We tested various properties of the two strains, included tolerance, cell adhesion, hemolytic activity, catalase activity, gelatin hydrolysis, arginine hydrolysis ability, bile salt hydrolysis capacity, mucin degradation, bioamine, D-/L-lactic acid production and antibiotic susceptibility, to confirm the safety of LC86 and LC89 both *in vitro* and *in vivo*. Additionally, we studied the acute toxicity of LC86 and LC89 in mice through a 14-day oral gavage experiment.

**Results:**

The two strains selected were identified as *Lacticaseibacillus paracasei* and *Lacticaseibacillus casei*. The genomes of both LC86 and LC89 were devoid of virulence, antibiotic resistance and pathogenicity genes. LC86 and LC89 exhibited good tolerance to temperature, artificial gastric fluid and artificial intestinal fluid; they were non-hemolytic, their catalase activity, gelatin hydrolysis, arginine hydrolysis and bile salt hydrolysis were all negative. They exhibited the capability to break down proteins and demonstrated sensitivity to a range of antibiotics. The oral LD_50_ for both LC86 and LC89 in mice was >2 × 10^10^ CFU/kg.

**Conclusion:**

The experimental results above demonstrated the probiotic characteristics and safety of LC86 and LC89, indicating their potential as candidates for probiotics for human and animal applications.

## 1 Introduction

The realm of probiotics is at the forefront of scientific research and commercial development due to their potential to modulate human health through the modulation of the gut microbiome. Probiotics are characterized as living microorganisms that, when ingested in sufficient quantities, bestow health advantages upon the host (Hill et al., 2014). It is recommended that daily consumption of between 10^8^ and 10^9^ Colony-Forming Units (CFU) per gram of probiotic bacteria enables these microbes to withstand the harsh conditions of ingestion and subsequently perform their beneficial physiological roles within the human body (Dimitrellou et al., 2019). Currently, the health consciousness of consumers worldwide is increasingly growing, leading to a rising demand for health foods and dietary supplements, which in turn drives the continuous expansion of the probiotics market. This trend prompts food researchers to continuously study the characteristics of probiotic powders and develop new probiotic products (Lu et al., 2021).

Commercially utilized in the food sector, probiotics, mainly *Lactococcus, Lactobacillus*, and *Bifidobacterium*, are live microbes that reside in the gut and exhibit a range of health benefits. They contribute to human gastrointestinal health by regulating the microbiota, which includes suppressing the proliferation of potentially harmful bacteria (Zheng et al., 2020). In particular, the excellent qualities of *Lacticaseibacillus paracasei* and *Lacticaseibacillus casei* are being increasingly explored by researchers. Genomic analysis uncovers that probiotic strains of *Lactobacillus* and *Bifidobacterium* possess safety attributes, exhibit antiviral capabilities (Qureshi et al., 2020) and contain factors that promote adherence to the host (Abdelhamid et al., 2019). *Lactobacillus* is a member of the human gut microbiota and has shown various health benefits for the host, including alleviating irritable bowel syndrome (JanssenDuijghuijsen et al., 2024), regulating immunity (Gai et al., 2023), reducing cholesterol (Sun et al., 2020), improving allergic rhinitis (Liu et al., 2022), balancing oral microbiota (Rui et al., 2024), preventing cancer (Eslami et al., 2019). *L. paracasei* and *L. casei*, two species within this genus, have garnered significant attention due to their reported health benefits, including immune modulation, mitigating muscle weakness (Cai et al., 2024), pathogen inhibition and enhancement of gut barrier function (Aktas et al., 2016). These species are naturally occurring in the human gastrointestinal tract and various fermented foods, contributing to their acceptance as safe for human consumption. However, the safety and efficacy of any potential probiotic strain must be rigorously evaluated before they can be considered for use in food products or dietary supplements. Currently, there are many widely accepted methods to evaluate the potential and safety of probiotics (Abe et al., 2010; Lara-Villoslada et al., 2009; Morovic et al., 2017). The characteristics of probiotics are primarily assessed through *in vitro* experiments, including tests for mucosal cell adhesion, tolerance to acid and bile salts, mucin degradation ability (Abe et al., 2010) and arginine hydrolysis capacity. The safety of probiotics can be verified through both *in vitro* and *in vivo* experiments, including genomic sequencing, hemolysis tests, antimicrobial resistance tests (Lara-Villoslada et al., 2009) and acute oral toxicity tests (Morovic et al., 2017).

This study evaluates the safety and potential of two probiotic strains, *Lacticaseibacillus paracasei* LC86 and *Lacticaseibacillus casei* LC89. *L. paracasei* LC86 is a strain with excellent properties isolated from traditional Chinese yogurt (Qinghai, China) and possesses independent intellectual property rights. It had been deposited with China General Microbiological Culture Collection Center (CGMCC No. 1.12731). *L. casei* LC89 is also a strain with independent intellectual property rights, isolated from traditional Chinese fermented foods (Qinghai, China), deposited in China General Microbiological Culture Collection Center (CGMCC No. 15409). In the present study, we embarked on a meticulous exploration of the safety and the potential of LC86 and LC89. The evaluation included identification of the strains, genetic safety assessment through whole genome sequencing, *in vitro* tests for physiological properties like acid and bile salt tolerance, an acute oral toxicity study in mice. The sequencing confirmed their species identity and absence of harmful genes. *In vitro* tests checked for tolerance, adhesion to intestinal cells and the absence of harmful activities like hemolysis and biogenic amine production. The strains' antibiotic susceptibility was also assessed. An *in vivo* study in mice evaluated their oral toxicity over 14 days. Strain identification is carried out to determine the species and characteristics of the strains, avoiding the use of unknown or harmful strains that may pose a threat to human health. Whole genome sequencing can identify the presence of genes that may be harmful to human health, such as toxin genes and antibiotic resistance genes. The acid and bile salt tolerance tests of strains are to assess their survival ability in the gastric acid and intestinal environment, which is particularly important for oral products. The adhesion test of strains evaluates whether they can adhere to intestinal cells, which is necessary for probiotics to colonize and function in the gut. Assessing the susceptibility of strains to different antibiotics is crucial for preventing the development of antibiotic resistance and ensuring the effectiveness of treatments. Evaluating the acute toxicity of strains in animal models predicts their potential toxicity and safety for human use.

In conclusion, the introduction of *L. paracasei* LC86 and *L. casei* LC89 as potential probiotic candidates necessitates a thorough safety evaluation. This study presents a comprehensive approach to assessing the safety of these strains, from genetic characterization to *in vivo* toxicity testing. The findings from this research will be instrumental in determining the suitability of LC86 and LC89 for use in food products and dietary supplements, contributing to the growing body of knowledge on the safety and application of probiotics in human health.

## 2 Materials and methods

### 2.1 Bacterial strains

Strains LC86 and LC89 were sourced from Wecare Probiotics Co., Ltd (Suzhou, China), cultivated under anaerobic conditions at 37°C using De Man-Rogosa-Sharpe medium (MRS, Qingdao Hi-Tech Industrial Park Hope Bio-Technology Co., Ltd, Qingdao, China). Two strains of *Lactobacillus* isolated from traditional yogurt and fermented foods were identified through a comprehensive analysis of cellular morphology, physiological and biochemical characteristics, 16S rRNA (Choudhary et al., 2019) gene sequences, *dnaK* gene sequences, and other experimental data, referring to the “Bergey's Manual of Systematic Bacteriology” and related research (2002). The two strains were identified as *Lacticaseibacillus paracasei* and *Lacticaseibacillus casei*, respectively. The strains were inoculated into a 25% (v/v) glycerol solution and stored at −80°C for preservation.

### 2.2 Whole genome sequencing and biochemical analysis

Employed a sophisticated nucleic acid extraction method to extract genomic DNA from LC86 and LC89, then sequenced them using the PacBio Sequel II and MGI G99 platform (Beijing Genomics Institute, Shenzhen, Guangdong, China). Predicted tRNA genes across the entire genome using tRNAscan-SE and predicted rRNA genes using Barrnap (http://www.vicbioinformatics.com/software.barrnap.shtml). The prediction of other non-coding RNAs was primarily obtained by comparison with the Rfam database; CRISPR elements were predicted using CRISPR finder, gene prediction for the whole genome sequence was performed using the GeneMarkS software. Performed average nucleotide identity (ANI) analysis using fastANI.

For an in-depth analysis of antibiotic resistance genes and virulence factors within the genomes, we aligned and screened all identified coding sequences (CDSs) using the following databases: the Comprehensive Antibiotic Resistance Database (CARD), ResFinder (on the foundation of the ARDB database), and AMRFinderPlus (NCBI Antimicrobial Resistance Gene Finder Plus). Additionally, we used ISEScan v.1.7.2.1 to identify the insertion sequence. In the screening for virulence factors, we conducted analysis using VirulenceFinder 2.0.5 (https://cge.food.dtu.dk/services/VirulenceFinder/) and the Virulence Factor Database (VFDB, http://www.mgc.ac.cn/VFs/). The VFDB, developed by the Chinese Academy of Medical Sciences, collects information on the composition, structure, function, pathogenesis, virulence islands, sequences and genomes of over 100 important pathogenic bacteria. Furthermore, we utilized the PathogenFinder 1.1 database (https://cge.food.dtu.dk/services/PathogenFinder/) to evaluate bacterial pathogenicity. We searched for biogenic amine synthesis genes on the LC86 and LC89 genome in the NR and KEGG databases, assessed genes/pathways related to the production of harmful metabolites in LC86 and LC89 through KEGG searches. Ultimately, to visually display the genome structure of LC86 and LC89, we generated circular genomic plots using cgview.

### 2.3 Tolerance to strains

Conducted tolerance tests on strains LC86 and LC89 for temperature, acidity, artificial gastric fluid, artificial intestinal fluid and bile salts. The acid pH of the MRS liquid medium was adjusted to 2.5, 3.0 using HCl (Zhang et al., 2022). The artificial gastric juice was prepared by dissolving 0.1 g of pepsin (1:10,000, Sigma, St. Louis, MO, USA) in 10 ml of NaCl solution (0.5%, w/v), adjusted the pH to 2.5, 3.0 with HCl, filter sterilize. Prepared artificial intestinal juice by dissolving 0.1 g of trypsin (Sigma, St. Louis, MO, USA) in 10 ml of a 0.5% (w/v) NaCl solution, adjusted the pH to 8.0 with NaOH, filter sterilize. To prepare bile salt, MRS broth contained 0.1% pig bile salt (Sigma-Aldrich Corp., St. Louis, MO, USA) and then sterilized.

Strains LC86 and LC89 were cultured in MRS medium and centrifuged at 4°C (10,000 × *g*, 10 min). The pellet was resuspended in MRS liquid medium and incubated at temperatures of 30, 37, 42, 45°C for 24 h. After incubation, the turbidity of the culture broth was observed. The supernatant was discarded, the pellet was resuspended in 10 ml of either acidic MRS liquid medium, artificial gastric juice, artificial intestinal juice, or bile salt solution. The mixtures were thoroughly vortexed, and the viable bacterial count was assessed by plating at time zero (0 h), establishing the initial count (N0) as the control. Subsequently, the mixtures were incubated at 37°C for 2 h (Chen et al., 2024), after which the viable bacterial count was determined again using the same plating method. The survival rate was calculated using the formula: Survival rate/% = lgNi/lgN0 × 100, where Ni represents the viable bacterial count after “I” h of treatment. This procedure was conducted in triplicate to ensure reliability of the results.

### 2.4 Cytotoxicity

The fermentation broth cytotoxicity and bacterial cell toxicity for LC86 and LC89 were assessed (Hardy et al., 2018). The bacterial cell cytotoxicity was assessed using the Lactate Dehydrogenase Cytotoxicity Assay Kit (Promega, USA), while the fermentation broth cytotoxicity was evaluated with the CCK-8 Assay Kit (TargetMol, USA). Seeded Caco-2 (Lee et al., 2024) cells at a density of 1.5 × 10^4^ cells per well in a 96-well plate to assess fermentation broth cytotoxicity. Centrifuged the bacterial fermentation liquid (5,000 × *g*, 10 min), then collected the supernatant, adjust the pH to 7.4 using 5 M NaOH, filtered it through a 0.22 μm filter. Performed the same procedure with fresh MRS medium. Prepared Minimum Essential Medium [MEM, Thermo Fisher Scientific (China) Co., Ltd, Shanghai, China] with 20% Fetal Bovine Serum [FBS, Thermo Fisher Scientific (China) Co., Ltd, Shanghai, China], supplemented with either 10% or 20% of the bacterial supernatant and also prepared MEM with 20% FBS containing 10% or 20% of the fresh MRS medium as a control. Replaced the Caco-2 cell culture medium with the prepared supernatant media and incubated for 24 h. After incubation, added the CCK-8 solution and incubated for an additional 3 h. Measured the optical density at 450 nm (OD_450_) to determine the cell viability. To evaluate Bacterial Cell Toxicity, began by seeding Caco-2 cells at a rate of 1.5 × 10^4^ cells per well in a 96-well plate. Next, centrifuged the bacterial suspension (5,000 × *g*, 10 min), then pelleted the bacterial cells and resuspend them in 1 ml of MEM medium containing 20% FBS. Created a series of dilutions to achieve bacterial suspensions with concentrations of 10^7^, 10^8^, 10^9^ CFU/ml. Prior to the assay, washed the Caco-2 cells with PBS to clear away the culture medium and any dead cells, repeating the wash twice. Subsequently, added 200 μl of each bacterial suspension to the wells containing the Caco-2 cells, with quadruplicate wells for each concentration, incubated under conditions of 37°C and 5% CO_2_. At intervals of 4 and 8 h post incubation, collected 50 μl of the culture supernatant from each group and assessed cell toxicity employing the LDH-Glo cytotoxicity assay kit, which utilizes a chemiluminescence detection method.

### 2.5 Cell adhesion

Determined the adhesive properties of LC86 and LC89 to Caco-2 cells (Lee et al., 2024). To assess the adhesive properties of LC86 and LC89 to Caco-2 cells, seeded the cells at a density of 3 × 10^5^ cells per well in a six-well plate. After centrifuging (5,000 × *g*, 10 min) the bacterial suspension, resuspended the pellet in 10 ml of MEM supplemented with 20% FBS (without antibiotics) and adjusted the concentration to 10^8^ CFU/ml. Washed the Caco-2 cells with PBS to remove the MEM medium, repeating the wash twice. Added 1 ml of the bacterial suspension to each well, with duplicates for each group and incubated at 37°C for 4 h. Use MEM with 20% FBS (without antibiotics) as a control. After incubation, washed the wells with Phosphate-Buffered Saline [PBS, Thermo Fisher Scientific (China) Co., Ltd, Shanghai, China] five times to remove non-adherent bacteria. Add 1 ml of 0.3% Triton-X100 lysis solution to each well, incubated at room temperature for 15 min, then added 1 ml of MRS liquid medium and mixed thoroughly. Performed serial dilutions of the lysate (1:100, 1:1,000) and spread 400 μl onto MRS agar plates. After anaerobic incubation at 37°C for 24 h, counted the digested Caco-2 cells in the control wells to determine the total cell count (*N*_c_). Counted the colonies on the MRS plates to calculate the total number of adhering bacteria (*N*_b_) using the formula: *N*_b_ = (average number of colonies) × (2 ml/0.4 ml) × (dilution factor). Finally, calculated the average number of probiotic bacteria adhering to every 100 Caco-2 cells as *N*_b_/*N*_c_ × 100.

### 2.6 Hemolytic activity

Strains LC86 and LC89 were inoculated on Columbia blood agar plates (Qingdao Hi-Tech Industrial Park Hope Bio-Technology Co., Ltd, Qingdao, China) containing 5% (w/v) fresh sheep blood and incubated at 37°C for 48 h. Subsequently, the plates were examined to determine if hemolysis rings were present. A viridans hemolysis ring indicated that an organism is α-hemolytic, whereas a transparent hemolysis ring indicated that it was β-hemolytic. The assay was conducted in triplicate on three separate occasions (Chen et al., 2024; Arellano et al., 2020).

### 2.7 Catalase activity

Performed the catalase test by taking a colony from the cultured strain and spreading it onto a microscope slide. Applied a drop of 3% hydrogen peroxide (H_2_O_2_, Sinopharm Chemical Regent Co., Ltd., Shanghai, China) to the colony. Observed for the production of bubbles. The presence of bubbles signified a positive catalase reaction, while their absence indicated a negative result.

### 2.8 Bile salt hydrolysis capacity

Prepared the MRS agar medium by incorporating 0.5% sodium deoxycholate, 0.2% sodium mercaptoacetate, 0.2% calcium chloride. After inoculating the test strain onto the plates, incubated them at 37°C under anaerobic conditions for 72 h to observe bile salt precipitation. The presence of precipitated bile salts indicated that the test strain possessed bile salt hydrolysis activity.

### 2.9 Gelatin hydrolysis

Inoculated the test strain into a gelatin biochemical tube and incubated it at 37°C under anaerobic conditions for 24 h. Following incubation, chill both the inoculated test tube and an uninoculated control tube at 4°C for 30 min. Then, assessed for gelatin liquefaction to determine the strain's activity.

### 2.10 Arginine hydrolysis ability

Subcultured the strain through two successive generations and streak-inoculated it onto Columbia blood agar plates. Transferred bacterial colonies to sterile saline to achieve a turbidity of ~3.75, which corresponds to a concentration of around 10^8^ CFU/ml, using a McFarland turbidity meter (Zhengzhou Bioda Instrument Co., Ltd., Henan, China) for calibration. Pipetted 2 drops (~0.06 ml) of the bacterial suspension into both arginine hydrolysis biochemical tubes and amino acid control biochemical tubes. Overlayed the medium's surface with 5–8 drops of sterilized liquid paraffin to prevent evaporation and oxidation. Incubated the tubes at 37°C for 72 h. Interpreted the results as follows: a blue-green color in the test tube and a yellow color in the control tube indicated a positive reaction. If both tubes turned yellow, the result was negative.

### 2.11 Mucin degradation

Prepared two batches of 50 CHL culture medium: one with 0.3% mucin (Sigma–Aldrich, Inc., MO, USA) and another without mucin. Inoculated each batch with a 1% bacterial suspension and incubated at 37°C under anaerobic conditions for 48 h. For analysis, took 75 μl of the culture liquid, mixed it with 25 μl of 4 × sample buffer, heated the mixture in a 100°C water bath for 5 min. Subsequently, performed SDS–PAGE electrophoresis and visualized the proteins by staining with Coomassie Brilliant Blue (Abe et al., 2010).

### 2.12 Bioamine

Prepared a series of standard solutions containing a mixture of biogenic amines (spermine, spermidine, cadaverine, putrescine, tryptamine, histamine) at concentrations ranging from 0 to 50.0 mg/L in increments of 5.0 mg/L, using 0.1 mol/L hydrochloric acid as the diluent. For the sample preparation, extracted the LC86/LC89 fermentation liquid with an equal volume of 10% (M/V) trichloroacetic acid for 1 h. Filter the mixture through double-layer filter paper and transferred 2 ml of the filtrate to a derivatization vial. To the vial, added 1 ml of 2 mol/L NaOH to adjust the pH to alkaline conditions. Then, added 10 μl of benzoyl chloride and derivatized the sample in a 30°C water bath for 20 min. To halt the derivatization, added 2 ml of saturated NaCl and heated the mixture at 60°C for 5 min. After cooling, added 3 ml of ether, shook well to mix, allowed the layers to separate. Transferred the upper organic phase to a clean test tube. Evaporated the ether under a stream of nitrogen to dryness and then dissolved the residue in 1 ml of methanol. Finally, injected 20 μl of this solution into the HPLC system to determine the biogenic amine content.

### 2.13 D-/L-lactic acid production capacity

Used the D-Lactate Enzyme Assay Kit (r-biopharm, Germany) and the D/L-Lactate Enzyme Assay Kit (r-biopharm, Germany) to test the ability of LC86 and LC89 to produce D-lactic acid and L-lactic acid, respectively. Performed the assays using the fermentation broth of LC86 and LC89. D-Lactic Acid: Added 100 μl of distilled water to 2,000 μl of reagent 1 to serve as the reagent blank. Added 100 μl of bacterial liquid to 2,000 μl of reagent 1 as the sample solution. Thoroughly mixed and incubated at 37°C for 3 min. Then measured the absorbance (A1). Subsequently, added 500 μl of reagent 2, mixed well again, incubated at 37°C for 10 min before measuring the absorbance (A2). D-/L-Lactic Acid: added 100 μl of distilled water to 2,000 μl of reagent 1 as the reagent blank. Added 100 μl of bacterial liquid to 2,000 μl of reagent 1 as the sample solution. After mixing well, incubated at 37°C for 1 min and measured the absorbance (A1). Then, added 500 μl of reagent 2, mixed thoroughly, incubated at 37°C for 10 min to measure the absorbance (A2). The experiment was conducted in triplicate and the average value was taken.

### 2.14 Antibiotic susceptibility

The antibiotic susceptibility of strains LC86 and LC89 was assessed in accordance with the European Food Safety Authority (EFSA) guidance (European Food Safety Authority, 2018), which encompass testing for eight antibiotics: ampicillin, gentamicin, kanamycin, streptomycin, erythromycin, clindamycin, tetracycline and chloramphenicol (Sigma-Aldrich Corp., St. Louis, MO, USA). As per the EFSA guidelines, testing for vancomycin sensitivity was not required. The bacterial solution was adjusted to a concentration of 3 × 10^5^ CFU/ml using saline solution. Antibiotics were then added at varying concentrations through a serial dilution process. The minimum inhibitory concentration (MIC) was identified as the lowest antibiotic concentration that completely inhibits bacterial growth, with no visible increase in cell numbers observed.

### 2.15 Acute oral toxicity test

The evaluation of the acute oral toxicity of *Lacticaseibacillus paracasei* LC86 and *Lacticaseibacillus casei* LC89 was conducted in accordance with the GB 15193.3-2014 [“Guobiao” (Chinese National Standard), National Food Safety Standard—Acute Oral Toxicity Test]. Selected 6-week-old SPF-grade ICR mice from Shanghai Shenchang Biotechnology Co., Ltd. (SCXK (Shanghai) 2021-0002, Shanghai, China), with a body weight of 18–22 g. The feed was provided by Jiangsu Xietong Pharmaceutical Bio-engineering Co., Ltd. [Su Feed Approval (2019) 01008, Nanjing, China], in compliance with the relevant provisions of GB/T 14924.1-2001 [“Guobiao” (Chinese National Standard), Laboratory animals—General quality standard for formula feeds]. This experiment was approved by the Animal Ethics Committee of Technical Center for Animal Plant and Food Inspection and Quarantine of Shanghai Customs [permit number: SYXK (Shanghai) 2019-0033]. All experimental mice were housed in an SPF animal room with free access to food, under a temperature of 20–22°C and a relative humidity of 45%−65%. Twenty mice (half male and half female, unmated and non-pregnant female mice) were all subjected to a 5-day period of environmental adaptation and quarantine observation in the experimental setting. The mice were fasted (with unrestricted access to water) for 6 h before the experiment. A single-limit method was used and the test substance (LC86, LC89) was administered orally at a dose of 2 × 10^10^ CFU/kg body weight (BW), with a maximum gavage volume of 20 ml/kg BW. The general condition, behavioral changes, signs of poisoning, mortality of the animals were observed twice daily. The animals were weighed once every 7 days. Gross necropsy and observation were conducted on animals that died from poisoning and on those that survived for 14 days post-exposure.

### 2.16 Statistical analysis

All data in this article were analyzed using SPSS 26.0 software and were represented as mean ± standard deviation (SD). Group differences were assessed utilizing one-way Analysis of Variance (ANOVA) to determine the presence of statistically significant variations across the study cohorts. If the ANOVA revealed significant effects, Tukey's Honestly Significant Difference (HSD) test was employed for pairwise comparisons between groups, allowing for the identification of specific inter-group disparities. The threshold for statistical significance was set at a *p*-value of < 0.05, which denoted that observed differences between the experimental and control groups were unlikely to occur by chance and are thus considered to be meaningful.

## 3 Results

### 3.1 Whole genome sequencing and biochemical analysis

**Figure 1:**
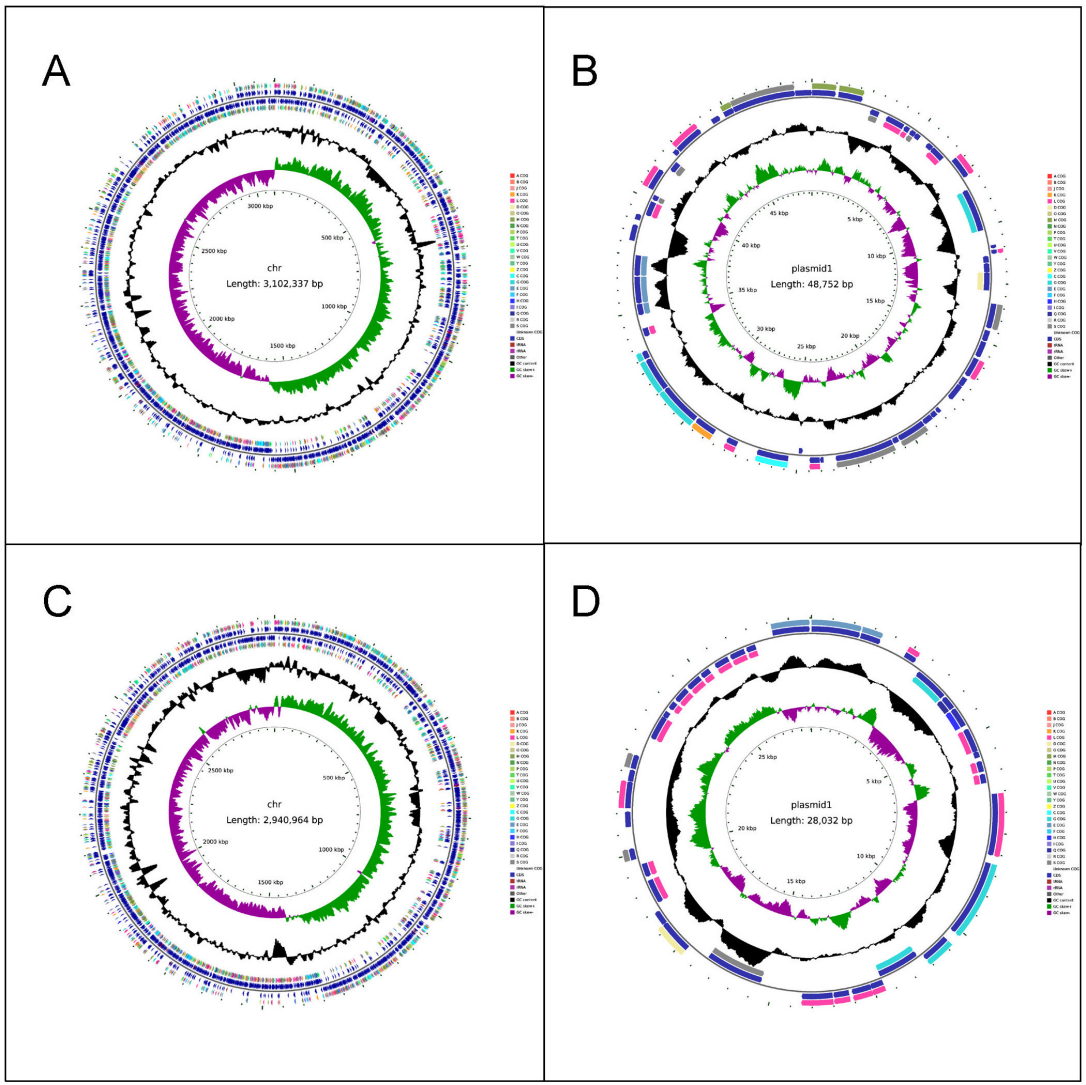
The completed genome maps of LC86 and LC89. **(A)** Chromosome circle map of LC86. **(B)** Plasmid circle map of LC86. **(C)** Chromosome circle map of LC89. **(D)** Plasmid circle map of LC89.

The chromosome length of LC86 was 3,102,337 base pairs (bp) and the plasmid length was 48,752 bp (Figures 1A, B). The complete genome GC content of LC86 was 46.34%, which was essentially consistent with the 46.5% GC content of the *L*. *paracasei* strains SMN-LBK, CBA3611, TCI727 and JCM 8130, as completed and sequenced in National Center for Biotechnology Information (NCBI). According to the analysis results of Average Nucleotide Identity (ANI), the ANI value between LC86 and *L*. *paracasei* SMN-LBK (whole genome accession number: GCF_024498315.1) was 99.99%, confirming this strain as *Lacticaseibacillus paracasei*, which was consistent with the results of bacterial identification. Using the AMRFinder, CARD and ResFinder databases for gene searches, no potential antibiotic resistance genes were detected in either the chromosome or the plasmid. Potential virulence-related genes were searched using the VirulenceFinder and VFDB databases, with no virulence-related genes identified in the chromosome or the plasmid. Pathogenicity gene analysis was conducted with the PathogenFinder database, confirming that this strain was non-pathogenic to humans. By searching the LC86 genome for biogenic amine synthesis genes using the KEGG and NR databases, the gene for ornithine decarboxylase was identified, located at positions 1,817,943–1,820,033 bp on the genome. Additionally, KEGG assessment of LC86 for genes related to the production of metabolic products indicates that LC86 did not contain bacterial toxins. LC86 did contain genes associated with the metabolic products of pyruvate, such as D-lactate dehydrogenase, and with the metabolic products of ornithine, such as ornithine decarboxylase. However, LC86 lacked genes related to the metabolic products of primary and secondary bile acids.

The chromosome length of LC89 was 2,940,964 bp, and the plasmid length was 28,032 bp (Figures 1C, D). The complete genome GC content of LC89 was 47.86%, which was essentially consistent with the 48% GC content of the *L*. *casei* strains ATCC393 and LC130, as completed and sequenced in NCBI. According to the analysis results of ANI, the ANI value between LC89 and *L*. *casei* ATCC393 [whole genome accession number: AP012544.1 (Chromosome), AP012545.1 (Plasmid 1), AP012546.1 (Plasmid 2)] was 99.95%, confirming this strain as *Lacticaseibacillus casei*, which was consistent with the results of bacterial identification. No antibiotic resistance or virulence genes were found in its genome. The strain was non-pathogenic to humans. LC89 had genes for ornithine decarboxylase and D-lactate dehydrogenase but lacked genes for primary and secondary bile acid metabolism. The gene for ornithine decarboxylase was identified, located at positions 1,600,679–1,602,769 bp on the genome.

### 3.2 Tolerance to strains

Strains LC86 and LC89 demonstrated survival rates higher than 95% after being cultured for 2 h in acidic environments (pH 2.5, 3.0) and in artificial gastric juice (pH 2.5, 3.0). Furthermore, the survival rate of LC86 in artificial intestinal juice and bile salts were 99.93% and 96.89%, respectively, while the survival rates for LC89 in artificial intestinal juice and bile salts were 98.42% and 99.90% (Table 1). These findings indicated that both LC86 and LC89 possessed strong tolerance to artificial gastric juice, artificial intestinal juice, and bile salts. The ability of strains LC86 and LC89 to grow at temperatures of 30, 37, 42, 45°C indicated that these two strains had a broad temperature adaptation range. Their temperature tolerance should have been taken into consideration during production and storage processes to ensure product quality and safety (Table 2).

**Table 1:** LC86 and LC89 simulated gastrointestinal conditions test results.

**Strain**	**Testing Index**		**lg CFU/mL**		**Bacterial survival rate/%**	
			**0 h**	**2 h**		
LC86	Acid	2.5	11.00 ± 0.012	10.531 ± 0.045	95.74
			3.0	11.00 ± 0.091	10.784 ± 0.057	98.04
	Artificial gastric juice	2.5	7.242 ± 0.083	7.120 ± 0.083	98.32
			3.0	7.111 ± 0.069	7.083 ± 0.057	99.62
	Artificial intestinal fluid		7.178 ± 0.056	7.173± 0.063	99.93
	Bile salt		11.00 ± 0.017	10.658 ± 0.013	96.89
LC89	Acid	2.5	11.05 ± 0.010	10.566 ± 0.006	95.62
			3.0	11.00 ± 0.006	10.767 ± 0.011	97.88
	Artificial gastric juice	2.5	7.271 ± 0.074	7.246 ± 0.075	99.66
			3.0	7.158 ± 0.059	7.141± 0.012	99.76
	Artificial intestinal fluid		7.058 ± 0.053	6.947 ± 0.058	98.42
	Bile salt		6.887 ± 0.050	6.881 ± 0.044	99.90

**Table 2:** LC86 and LC89 temperature tolerance test results.

**Strain**	**Cultivation temperature**	**30°C**	**37°C**	**42°C**	**45°C**
LC86	Growth state	+	+	+	+
LC89	Growth state	+	+	+	+

“+” indicates that the culture medium is turbid and bacterial growth is observed.

### 3.3 Cytotoxicity

**Figure 2:**
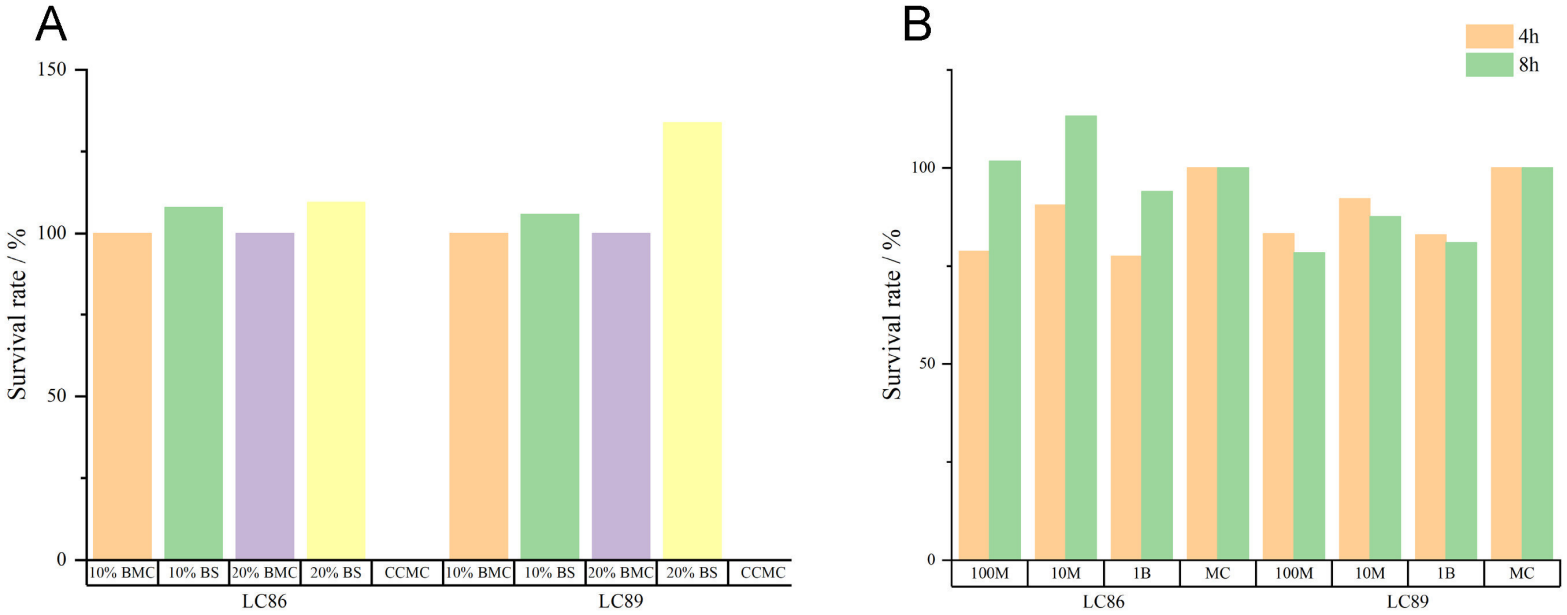
Cytotoxicity test results of LC89 and LC89 fermentation broth and bacterial cells. **(A)** Cytotoxicity test results of LC89 and LC89 fermentation broth. CCMC, cell culture medium control; 10% BMC, 10% bacterial medium control; 20%BMC, 20% bacterial medium control; 10%BS, 10% bacterial supernatant; 20%BS, 20% bacterial supernatant. **(B)** Cytotoxicity test results of LC89 and LC89 bacterial cells. M, million; B, billion; MC, medium control.

The experimental data indicated that Caco-2 cells cultured in 10% or 20% fermentation broth of the LC86 and LC89 bacterial strains for 3 h showed good survival, when Caco-2 cells were co-cultured with the bacterial cells of LC86 and LC89 at three different concentrations (10^7^, 10^8^, 10^9^ CFU/ml) for 4 and 8 h, there was no significant difference compared to the control group cultured in the medium alone (*P* > 0.05); this suggested that the bacterial cells and fermentation broth of LC86 and LC89 were non-toxic to Caco-2 cells (Figure 2).

### 3.4 Cell adhesion

The cell adhesion assay results showed that the total cell count was *N*_c_ = 6.365 × 10^4^ CFU. For the adhered bacteria, the count was *N*_b_ = 38,250 CFU for LC86 and *N*_b_ = 19,750 CFU for LC89. The amount of LC86 bacteria adhering to every 100 cells was *N*_b_/*N*_c_ × 100 CFU = 60.09 CFU. Similarly, the amount of LC89 bacteria adhering to every 100 cells was *N*_b_/*N*_c_ × 100 CFU = 31.03 CFU. The study above demonstrated that LC86 and LC89 had good adhesive properties to Caco-2 cells. LC89 exhibited slightly lower adhesion ability to Caco-2 cells compared to LC86.

### 3.5 Hemolytic activity

**Figure 3:**
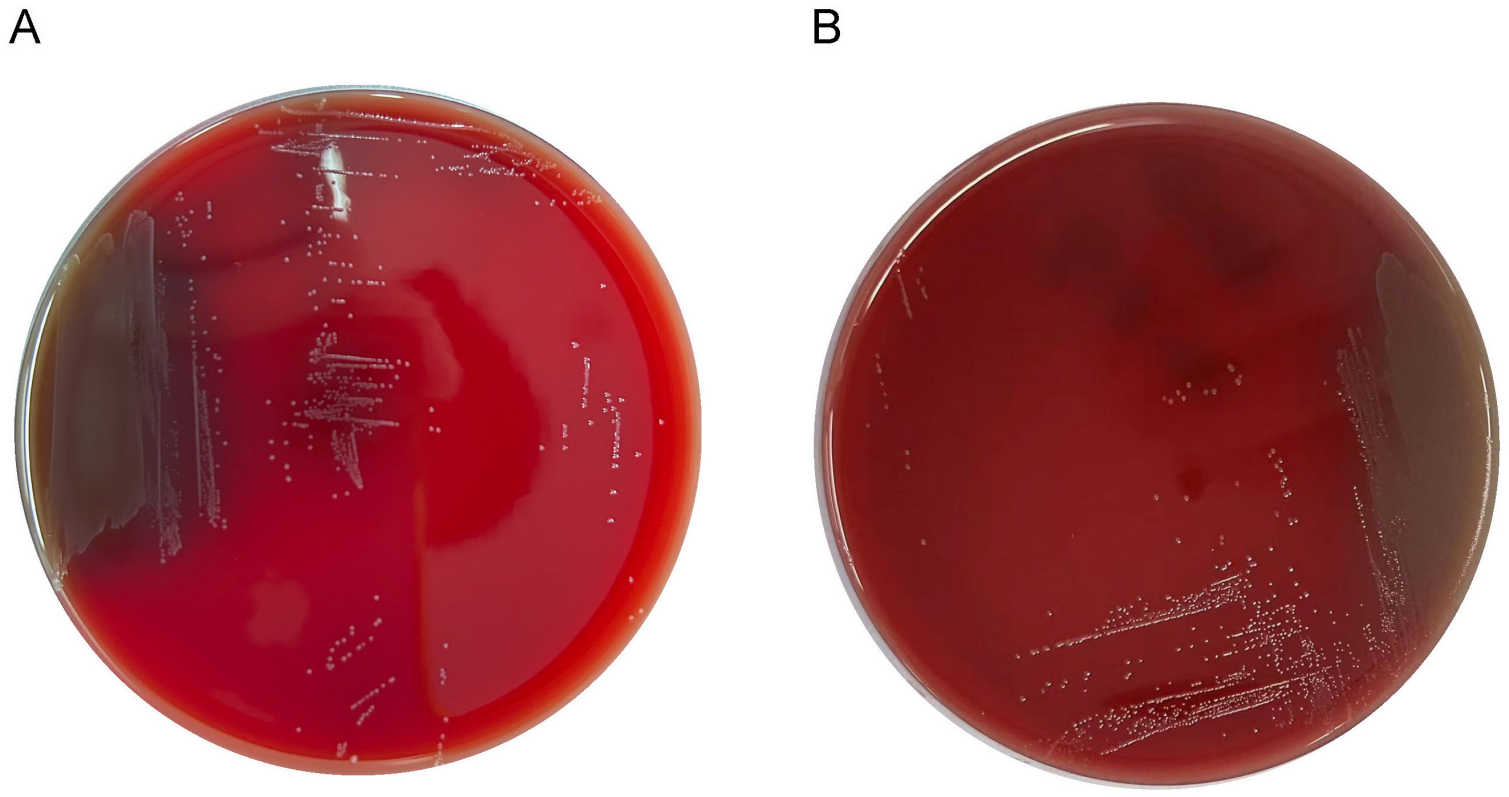
Growth of LC86 and LC89 on blood plate culture medium. **(A)** Growth of LC86 on blood plate culture medium. **(B)** Growth of LC89 on blood plate culture medium.

No hemolytic zones were observed around the LC86 and LC89 colonies grown on Columbia blood agar plates, indicating hemolytic negativity (Figure 3).

### 3.6 Catalase activity

Under the conditions of this test, catalase activity was negative in LC86 and LC89.

### 3.7 Bile salt hydrolysis capacity

**Figure 4:**
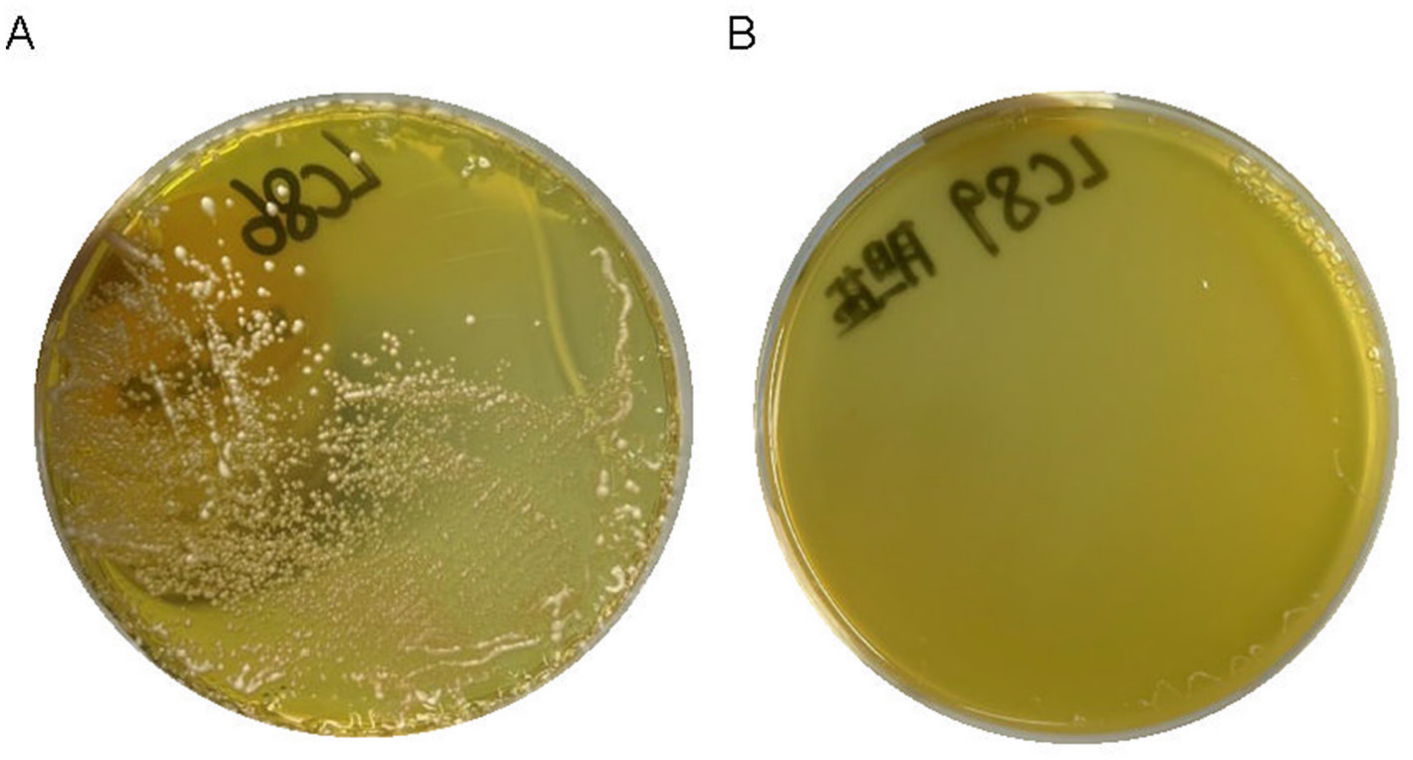
The result of white bile salts precipitate around bacteria growing on the culture medium. **(A)** The result of LC86 growing on a medium with white bile salts, showing precipitation around the colonies. **(B)** The result of LC89 growing on a medium with white bile salts, showing precipitation around the colonies.

The precipitation of white bile salts around the bacteria growing on the medium indicated that strains LC86 and LC89 possess bile salt hydrolysis capability (Figure 4).

### 3.8 Gelatin hydrolysis

Under the conditions of this test, LC86 and LC 89 were negative for gelatin hydrolysis.

### 3.9 Arginine hydrolysis ability

After the experiment, it was observed that both the test tube and the control tube turned yellow, indicating that LC86 and LC89 showed a negative result for arginine hydrolysis.

### 3.10 Mucin degradation

The experimental results showed that, compared to the uninoculated medium, LC86 and LC89 did not reduce the mucin zone. This indicated that LC86 and LC89 did not have mucin degradation activity.

### 3.11 Bioamine

The concentrations of tryptamine, putrescine, cadaverine and spermidine in the LC86 fermentation liquid were 2.73, 1.59, 1.98, 2.30 mg/L, respectively. Histamine and spermine were not detected, with concentrations below the detection limit of the method (1 mg/L). In the LC89 fermentation liquid, the concentrations of putrescine, cadaverine, and spermidine were measured at 1.38, 1.85, 2.37 mg/L, respectively. The absence of tryptamine, histamine, spermine suggested that their levels were below the detection limit of the method (1 mg/L).

### 3.12 D-/L-lactic acid production capacity

The L-lactic acid content in the fermentation broth of LC86 and LC89 was 0.96 and 0.891 g/L, respectively, while the D-lactic acid content was 0.023 and 0.035 g/L, respectively. The D-lactic acid content of both strains was significantly lower than that of L-lactic acid (Supplementary Table S1).

### 3.13 Antibiotic susceptibility

The minimum inhibitory concentrations (MICs) of various antibiotics against LC86 were found to be 0.5 μg/ml for ampicillin, 8 μg/ml for gentamicin, 32 μg/ml for kanamycin, 32 μg/ml for streptomycin, 0.125 μg/ml for erythromycin, 0.125 μg/ml for clindamycin, 0.5 μg/ml for tetracycline, 2 μg/ml for chloramphenicol. The MIC of LC89-Ampicillin was 0.032 μg/ml, LC89-Kanamycin was 32 μg/ml, LC89-Gentamicin was 8 μg/ml, LC89-Streptomycin was 16 μg/ml, LC89-Erythromycin was 0.032 μg/ml, LC89-Clindamycin was 16 μg/ml, LC89-Tetracycline was 1 μg/ml, LC89-Chloramphenicol was 2 μg/ml (Supplementary Table S2). The determined MICs for LC86 and LC89 were at or below the species-specific breakpoints defined by the EFSA guidance, demonstrated that the strain is susceptible to pertinent antibiotics.

### 3.14 Acute oral toxicity test

During the 14-day acute oral toxicity study in mice, no signs of poisoning or mortality were observed in the test animals. There were no abnormal changes in weight gain for either male or female animals. At the end of the observation period, a gross pathological dissection was performed on the test animals, and no pathological changes were found in any organs. The oral LD_50_ of both LC86 and LC89 in mice were >2 × 10^10^ CFU/kg (Table 3).

**Table 3:** Acute oral toxicity test results of *Lacticaseibacillus paracasei* LC86 and *Lacticaseibacillus casei* LC89 in mice.

**Test strain**	**Dose/(CFU/kg BW)**	**Gender**	**No. of animals/each**	**Weight/g**			**No. of death animals/each**
				**0 day**	**7 days**	**14 days**	
LC86	2 × 10^10^	♀	10	18.4 ± 0.52	24.5 ± 0.85	29.2 ± 0.92	0
		♂	10	18.3 ± 0.48	26.9 ± 0.74	34.4 ± 0.70	0
LC89	2 × 10^10^	♀	10	18.5 ± 1.53	24.1 ± 0.74	28.9 ± 0.88	0
		♂	10	18.2 ± 0.42	25.7 ± 0.67	34.1 ± 0.99	0

## 4 Discussion

Based on the core genome phylogeny, conserved average amino acid identity, strain-specific characteristic genes, physiological criteria, and bio-ecological studies, *Lacticaseibacillus casei, Lacticaseibacillus paracasei* and *Lacticaseibacillus rhamnosus* were all classified under the *Lacticaseibacillus* (Zheng et al., 2020). Due to their commercial, industrial and health application potential, they were among the most studied species (Hill et al., 2018). *L. casei* and *L. paracasei* were commonly found in the gastrointestinal tracts of humans and animals. Recognized for their safety, these species were included in the EFSA Qualified Presumption of Safety (QPS) list and were also endorsed in the “List of Microorganisms Suitable for Use in Food” by the National Health Commission of China. Despite their recognized status, we conducted comprehensive testing, included genetic safety, physiological characteristics, acute oral toxicity of strains *L. paracasei* LC86 and *L*. *casei* LC89, to ensure their safety for human consumption.

For a *Lactobacillus* strain to attain potential probiotic status, it was essential to tolerate the harsh gastrointestinal environment (Ren et al., 2014). In order to reach the host's small intestine, the first challenge was the acidic environment of the stomach, where gastric juice had a pH value generally < 2; however, ingested food had a buffering effect, which typically raised the pH to 3, thus a pH of 3 is generally considered the optimal pH for the survival of probiotics (Liu et al., 2013). Both LC86 and LC89 demonstrated good survival in pH = 3 conditions and artificial gastric juice. Concurrently, bile salts in the small intestine could adversely affect the viability of probiotics (Rupa and Mine, 2012). However, LC86 and LC89 also exhibited excellent tolerance to bile salts. The temperature tolerance results indicated that both strains could adapt to a range of temperatures, which was essential for their viability during production, storage and consumption. *L. paracasei* ZEM54 exhibited good tolerance to simulated gastric juice (pH = 3), 0.3% bile salts, 10% NaCl salt solution and 37°C (Qureshi et al., 2020). *L. casei* KGC1201 had good acid resistance and bile salt tolerance (Lee et al., 2023). These results clearly demonstrated their potential for use in nutritional applications. The adhesive capacity of probiotic strains was considered a key factor in enhancing their health benefits (Maryam et al., 2021), as their ability to adhere to the host's intestinal tract facilitates extended survival in the gastrointestinal tract (GIT) and promotes interactions between the bacteria and the host (Li et al., 2020). Therefore, the ability of probiotics to adhere to intestinal epithelium was crucial. To ensure the safety of the tested probiotic strains, their cytotoxicity in Caco-2 cells was assessed before the adhesion capacity test (Lee et al., 2024). Both LC86 and LC89 exhibited no toxicity at concentrations up to 1 × 10^9^ CFU/ml in Caco-2 cells. Consequently, the probiotic strains at a concentration of 1 × 10^8^ CFU/ml were used for the adhesion assay. LC86 and LC89 demonstrated good adhesive properties. These findings were consistent with many researchers' results, including the strong binding capacity of commercial probiotic strains of *Lactobacillus* and *Bifidobacterium* to Caco-2 and HT-29 cells (Lee and Kang, 2022; Patrone et al., 2021; Gotteland et al., 2014).

The use of genomics had facilitated the understanding of the molecular mechanisms behind probiotic-related characteristics. Genomic analysis also provided an in-depth look at the functional mechanisms of potential probiotic strains and their adaptability to the environment (Qureshi et al., 2020). The effects of probiotics were strain-specific, therefore, for newly isolated probiotic strains, it was necessary to conduct genomic sequencing and analysis. In this study, LC86 and LC89 underwent whole-genome sequencing and analysis. The results showed that LC86 and LC89 had not detect resistance genes, virulence genes, and pathogenicity genes. Studies had shown that there were no antibiotic resistance genes, virulence genes, or genes associated with antibiotic resistance detected in the genome of *L. casei* KGC1201 (Lee et al., 2023). Similarly, the safety assessment results of the *L. paracasei* PS23 genome showed that, when compared with the latest databases of antibiotic resistance genes and virulence factors, no genes with high similarity were found to be suspicious. Mice were fed different doses of PS23 for 28 days, and no adverse reactions were observed, indicating its safety (Li et al., 2023). This genetic safety profile, combined with the absence of adverse reactions in animal studies, supports further research on PS23 in various populations. There had been some clinical studies on PS23, and no adverse reactions were observed in both elderly individuals with sarcopenia and college athletes during and after the trial (Rondanelli et al., 2022; Wu et al., 2024). However, the ornithine decarboxylase gene was identified in the genome of LC86 and LC89. The gene was homologous to the known ornithine decarboxylase [EC: 4.1.1.17] and was possibly involved in the biosynthesis of putrescine. The sequence of this enzyme was widely present in the genomes of many *Lacticaseibacillus paracasei* strains in public domain databases (Li et al., 2023). Biogenice amines were basic nitrogenous compounds mainly generated by microbial decarboxylation of amino acids (Silla Santos, 1996). These molecules could have toxic effects on a host physiology such as hypertension, headache, nausea, diarrhea and fatal outcome in extreme cases (Suzzi and Torriani, 2015). To address safety concerns, the contents of histamine, putrescine, cadaverine, spermidine, histamine and spermine in the fermentation broths of LC86 and LC89 were detected. The results of this study indicated that the production levels of LC86 and LC89 were within the safe range. Histamine and spermine were not detected in the fermentation broth of LC86, and histamine, cadaverine, and spermine were not detected in the fermentation broth of LC89. The complete genome sequence of PS23 was compared with any existing biogenic amine-producing genes in the BLAST database, including histidine decarboxylase, tyrosine decarboxylase, ornithine decarboxylase, agmatine deiminase, lysine decarboxylase; it did not exhibit ornithine decarboxylase activity *in vitro* (Li et al., 2023).

Considering that hemolytic activity induced the lysis of red blood cells and the destruction of hemoglobin, leading to anemia, fever, and rash (Bang et al., 2021), we assessed the hemolytic properties of LC86 and LC89. No hemolytic zones were observed around the colonies of either strain grown on Columbia blood agar plates, indicating the absence of hemolytic activity. The bile salt hydrolysis capacity of LC86 and LC89, as evidenced by the precipitation of white bile salts around the colonies, suggested that these strains can modulate bile acid metabolism in the host. This ability is often associated with health benefits such as cholesterol reduction and improved lipid metabolism (Patel et al., 2010). The study had only conducted *in vitro* experiments, and further animal trials or even clinical studies could have been carried out to verify its effects on cholesterol reduction. Combining *in vivo* and *in vitro* tests would jointly validate the probiotic functions of their strains. The negative results for arginine hydrolysis and mucin degradation indicated that these strains do not produce enzymes that could potentially harm the host's tissues or disrupt the mucosal barrier. This was consistent with the reported findings on *Bifidobacterium breve, Bifidobacterium longum* subsp. *infantis, Lacticaseibacillus rhamnosus*, other such strains (Abe et al., 2010; Zhou et al., 2001; Fernández et al., 2005). This was an important consideration, as such activities could lead to adverse effects on the host. Biogenic amines, which were alkaline nitrogenous compounds primarily produced through the microbial decarboxylation of amino acids (Silla Santos, 1996), could exert toxic effects on a host physiology. Excessive intake could lead to symptoms such as hypertension, headaches, nausea, diarrhea and in severe cases could be life-threatening (Suzzi and Torriani, 2015; Shin et al., 2021). Lactic acid bacteria produce lactic acid in two optical isomer forms (L-type and D-type). Some lactic acid bacteria, included genera such as *Lactobacillus, Lacticaseibacillus* and *Bifidobacterium*, could produce both D-lactic acid and L-lactic acid (Stiles and Holzapfel, 1997). D-lactic acid was not metabolized in the human gut, its accumulation (Petersen, 2005) could potentially have led to health issues (Pohanka, 2020) due to the body's limited capacity to metabolize D-lactic acid. In this study, strains LC86 and LC89 were found to primarily produce L-lactic acid rather than D-lactic acid. Both LC86 and LC89 exhibited antibiotic resistance levels that did not exceed the breakpoints defined by the EFSA (European Food Safety Authority, 2018). The antibiotic susceptibility results confirmed that both strains were sensitive to a range of relevant antibiotics, suggested that they would not contribute to the spread of antibiotic resistance if used as probiotics. The research findings of Qureshi et al. (2020) indicated that no antibiotic resistance was observed in *L. paracasei* ZFM54; IDCC3451 did not have any resistance genes identified in its genomic sequence (Shin et al., 2021); PS23 exhibited MIC values lower than the MIC breakpoint values recommended by EFSA for the antibiotics tested (ampicillin, gentamicin, kanamycin, streptomycin, erythromycin, clindamycin, tetracycline, and chloramphenicol) (Li et al., 2023); this was consistent with the results of this study. With the continuous increase in the variety of antibiotics and the growing complexity of bacterial resistance issues, the scope of antibiotic testing can be expanded. Numerous strains of lactobacilli were naturally resistant to vancomycin (de Souza et al., 2019). The vancomycin resistance genes of *Lactobacillus* species appeared to be chromosomally located and were not easily transferable to other genera (Billot-Klein et al., 1994). While *in vitro* assessments of virulence characteristics were essential for probiotic candidate strains, *in vivo* studies using suitable animal models were crucial for confirming their safety (Lu et al., 2021). Oral toxicity studies were recognized as a standard approach for establishing the safety profile of a bacterial strain (Khalkhali and Mojgani, 2018). Previous studies had examined the acute oral toxicity of a range of *Lacticaseibacillus* and *Bifidobacterium* strains (Morovic et al., 2017; Zhang et al., 2013; Nataraj et al., 2024). No signs of acute toxicity were observed at the tested dose, which was consistent with the research findings of Qureshi et al. (2020) on *Lacticaseibacillus paracasei* ZFM54 and Lazarenko et al. (2021) on *Lacticaseibacillus casei* IMV B-7280.

In conclusion, the collective results of the genetic, physiological, toxicological assessments provide a robust foundation for the safety of *Lacticaseibacillus paracasei* LC86 and *Lacticaseibacillus casei* LC89. The strains exhibited a favorable safety profile, with no evidence of harmful genetic traits, physiological properties that were consistent with probiotic functionality, no acute toxic effects in the animal model. These findings explored the potential health benefits of these strains and their application in probiotic products.

However, despite the positive results achieved by this study, it had some limitations. First, safety experiments with rats were not conducted. This limited our understanding of the efficacy and safety of LC86 and LC89 in biological systems, as results from rats are more closely aligned with human biology compared to those from mice. Therefore, experiments conducted in rats would have been superior to those in mice in assessing the physiological effects and potential side effects of LC86 and LC89. Sub-acute, sub-chronic, or chronic tests should have been carried out using rats to provide information closer to human biological characteristics and to better assess the physiological effects and potential side effects of the strain. Second, genetic toxicity, reproductive toxicity, and carcinogenicity tests were not performed. These tests are crucial for assessing the potential impacts of the bacteria on the reproductive system and genome, respectively. Clinical studies on LC86 and LC89 in humans can also be conducted to further validate the safety and beneficial functions of LC86 and LC89. This would provide stronger evidence for the addition of these two strains to food products. Despite these limitations, the current study provided a solid foundation for the consideration of LC86 and LC89 as safe probiotic candidates.

In this study, a series of *in vitro* and *in vivo* experiments, included Whole Genome Sequencing, tolerance, cell adhesion, hemolytic activity, catalase activity, gelatin hydrolysis, arginine hydrolysis ability, bile salt hydrolysis capacity, mucin degradation, bioamine production, D-/L-lactic acid levels, antibiotic susceptibility and acute oral toxicity, were conducted to validate the safety of LC86 and LC89. This study laid the foundation for the application of these two strains in the food industry.

## Data Availability

The whole genome sequence of *Lacticaseibacillus casei* LC89 has been uploaded to NCBI (https://www.ncbi.nlm.nih.gov/), with the accession number 15003209. And the whole genome sequence of *Lacticaseibacillus paracasei* LC86 has been uploaded to NCBI (https://www.ncbi.nlm.nih.gov/), with the accession number 15003111.
