# Supplementary material for: Genome sequence and evaluation of safety and probiotic potential of Lacticaseibacillus paracasei LC86 and Lacticaseibacillus casei LC89
Source: Front Microbiol. 2025 Jan 27;15:1501502. doi: 10.3389/fmicb.2024.1501502 (PMC11808145; doi:10.3389/fmicb.2024.1501502)
Supplement: Supplementary file 1 [file Data_Sheet_1.docx]

**Supplementary Table1.** D-lactic acid and L-lactic acid content in the fermentation broth of LC86 and LC89. C_L-lactic acid_= C_D/L-lactic acid_ - C_D-lactic acid._

| Test No. | Group | D-lactic acid | | | | | D/L-lactic acid | | | | |
| --- | --- | --- | --- | --- | --- | --- | --- | --- | --- | --- | --- |
|  |  | A_1_ | A_2_ | A_2_-df×A_1_ | △A | C_D-lactic acid_ (g/L) | A_1_ | A_2_ | A_2_-df×A_1_ | △A | C_D/L-lactic acid_ (g/L) |
| 1 | Blank | 0.041 | 0.355 | 0.322 | / | / | 0.039 | 0.383 | 0.351 | / | / |
|  | LC86 | 0.179 | 0.502 | 0.357 | 0.035 | 0.013 | 0.212 | 3.178 | 3.007 | 2.655 | 0.987 |
|  | LC89 | 0.147 | 0.531 | 0.412 | 0.090 | 0.034 | 0.146 | 2.978 | 2.860 | 2.509 | 0.933 |
| 2 | Blank | 0.045 | 0.363 | 0.327 | / | / | 0.041 | 0.387 | 0.354 | / | / |
|  | LC86 | 0.185 | 0.528 | 0.379 | 0.052 | 0.019 | 0.224 | 3.165 | 2.984 | 2.630 | 0.978 |
|  | LC89 | 0.155 | 0.545 | 0.420 | 0.093 | 0.035 | 0.152 | 2.898 | 2.775 | 2.421 | 0.900 |
| 3 | Blank | 0.048 | 0.351 | 0.312 | / | / | 0.045 | 0.397 | 0.3606 | / | / |
|  | LC86 | 0.189 | 0.563 | 0.410 | 0.098 | 0.036 | 0.219 | 3.182 | 3.005 | 2.644 | 0.983 |
|  | LC89 | 0.149 | 0.529 | 0.409 | 0.096 | 0.036 | 0.150 | 3.021 | 2.900 | 2.539 | 0.944 |
| Mean and standard deviation | LC86 | / | / | / | / | 0.023±0.010 | / | / | / | / | 0.983±0.004 |
|  | LC89 | / | / | / | / | 0.035±0.001 | / | / | / | / | 0.926±0.019 |

**Supplementary Table2.** Minimum Inhibitory Concentrations (µg/mL) of Antibiotics for LC86 and LC89

| Strain | Group | 1 | 2 | 3 | 4 | 5 | 6 | 7 | 8 | 9 | 10 | 11 | 12 | 13 | 14 |
| --- | --- | --- | --- | --- | --- | --- | --- | --- | --- | --- | --- | --- | --- | --- | --- |
| LC86 | Ampicillin | 16- | 8- | 4- | 2- | 1- | 0.5- | 0.25+ | 0.125+ | 0.064+ | 0.032+ | 0.016+ | 0.008+ | 0.004+ | N- |
|  | Gentamicin | 256- | 128- | 64- | 32- | 16- | 8- | 4+ | 2+ | 1+ | 0.5+ | 0.25+ | 0.125+ | 0.064+ | N- |
|  | Kanamycin | 64- | 32- | 16+ | 8+ | 4+ | 2+ | 1+ | 0.5+ | 0.25+ | 0.125+ | 0.064+ | 0.032+ | 0.016+ | N- |
|  | Streptomycin | 32- | 16+ | 8+ | 4+ | 2+ | 1+ | 0.5+ | 0.25+ | 0.125+ | 0.064+ | 0.032+ | 0.016+ | 0.008+ | N- |
|  | Erythromycin | 8- | 4- | 2- | 1- | 0.5- | 0.25- | 0.125- | 0.064+ | 0.032+ | 0.016+ | 0.008+ | 0.004+ | 0.002+ | N- |
|  | Clindamycin | 16- | 8- | 4- | 2- | 1- | 0.5- | 0.25- | 0.125- | 0.064+ | 0.032+ | 0.016+ | 0.008+ | 0.004+ | N- |
|  | Tetracycline | 2- | 1- | 0.5- | 0.25+ | 0.125+ | 0.064+ | 0.032+ | 0.016+ | 0.008+ | 0.004+ | 0.002+ | 0.001+ | 0.0005+ | N- |
|  | Chloramphenicol | 64- | 32- | 16- | 8- | 4- | 2- | 1+ | 0.5+ | 0.25+ | 0.125+ | 0.064+ | 0.032+ | 0.016+ | N- |
| LC89 | Ampicillin | 16- | 8- | 4- | 2- | 1- | 0.5- | 0.25- | 0.125- | 0.064- | 0.032- | 0.016+ | 0.008+ | 0.004+ | N- |
|  | Kanamycin | 128- | 64- | 32- | 16+ | 8+ | 4+ | 2+ | 1+ | 0.5+ | 0.25+ | 0.125+ | 0.064+ | 0.032+ | N- |
|  | Gentamicin | 256- | 128- | 64- | 32- | 16- | 8- | 4+ | 2+ | 1+ | 0.5+ | 0.25+ | 0.125+ | 0.064+ | N- |
|  | Streptomycin | 32- | 16- | 8+ | 4+ | 2+ | 1+ | 0.5+ | 0.25+ | 0.125+ | 0.064+ | 0.032+ | 0.016+ | 0.008+ | N- |
|  | Erythromycin | 8- | 4- | 2- | 1- | 0.5- | 0.25- | 0.125- | 0.064- | 0.032- | 0.016+ | 0.008+ | 0.004+ | 0.002+ | N- |
|  | Clindamycin | 16- | 8+ | 4+ | 2+ | 1+ | 0.5+ | 0.25+ | 0.125+ | 0.064+ | 0.032+ | 0.016+ | 0.008+ | 0.004+ | N- |
|  | Tetracycline | 2- | 1- | 0.5+ | 0.25+ | 0.125+ | 0.064+ | 0.032+ | 0.016+ | 0.008+ | 0.004+ | 0.002+ | 0.001+ | 0.0005+ | N- |
|  | Chloramphenicol | 64- | 32- | 16- | 8- | 4- | 2- | 1+ | 0.5+ | 0.25+ | 0.125+ | 0.064+ | 0.032+ | 0.016+ | N- |
